# Intensivist supervision of resident-placed central venous catheters decreases the incidence of catheter-related blood stream infections

**DOI:** 10.1186/1754-9493-2-11

**Published:** 2008-04-30

**Authors:** Thomas J Papadimos, Sandra J Hensely, Joan M Duggan, James P Hofmann, Sadik A Khuder, Marilyn J Borst, John J Fath

**Affiliations:** 1 Department of Anesthesiology, University of Toledo College of Medicine, 3000 Arlington Avenue, Toledo, USA; 2 Infection Control Department, University of Toledo College of Medicine, 3000 Arlington Avenue, Toledo, USA; 3 Department of Medicine, University of Toledo College of Medicine, 3000 Arlington Avenue, Toledo, USA; 4 Department of Surgery, University of Toledo College of Medicine, 3000 Arlington Avenue, Toledo, USA

## Abstract

Catheter-related blood stream infections (CRBSI) cause significant morbidity and mortality. A retrospective study of a performance improvement project in our teaching hospital's surgical intensive care unit (SICU) showed that intensivist supervision was important in reinforcing maximal sterile barriers (MSB) use during the placement of a central venous catheter (CVC) in the prevention of CRBSI. A historical control period, 1 January 2001–31 December 2003, was established for comparison. From 1 January 2003–31 December 2007, MSB use for central venous line placement was mandated for all operators. However, in 2003 there was no intensivist supervision of CVC placements in the SICU. The use of MSB alone did not cause a significant change in the CRBSI rate in the first year of the project, but close supervision by an intensivist in years 2004–2007, in conjunction with MSB use, demonstrated a significant drop in the CRBSI rate when compared to the years before intensivist supervision (2001–2003), p < .0001. A time series analysis comparing monthly rates of CRBSI (2001–2007) also revealed a significant downward trend, p = .028. Additionally, in the first year of the mandated MSB use (2003), 85 independently observed resident-placed CVCs demonstrated that breaks in sterile technique (34/85), as compared those placements that had no breaks in technique (51/85), had more CRBSI, 6/34 (17.6%) vs. 1/51 (1.9%), p < .01. Interventions to reduce CRBSI in our SICU needed emphasis on adequate supervision of trainees in CVC placement, in addition to use of MSB, to effect lower CRBSI rates.

## Findings

Catheter-related blood stream infections (CRBSI) are associated with significant morbidity, mortality and cost [1-3]. Central venous catheter (CVC) use is important in the intensive care unit (ICU) for delivery of fluids and medications. There are over 15 million central catheter days per year in the USA with a CRBSI incidence of 4.3/1000 catheter-days; CVC related infections may be involved in 2400 to 20,000 deaths and over two billion dollars in costs annually [4-6]. The attributable costs of CRBSI have been determined to be $11,971, and are accompanied by extended ICU and hospital lengths of stay of 2.41 and 7.54 days, respectively [3].

The use of maximal sterile barriers (MSB) may reduce the CRBSI incidence. However, education of residents in proper CVC insertion techniques must also be emphasized. Use of MSB under the supervision of intensivists may synergistically decrease CRBSI incidence. Here we report the effect of MSB, before and after intensivist supervision of CVCs placed by residents, on CRBSI incidence in a surgical intensive care unit (SICU). The project was undertaken because our CRBSI rate from 1 November 2001–30 April 2002 was 10.2/1000 catheter-days (15 infections/1469 catheter-days), which retrospectively exceeded the 2002 National Nosocomial Infection Surveillance benchmark of 5.4/1000 catheter-days [5].

A performance improvement project was instituted in a 10 bed SICU that was part of a 319 bed, Trauma Level 1, university teaching hospital that averaged 667 admissions per year. Adult patients from all surgical subspecialties were admitted, including trauma, neurosurgery, cardiothoracic, urology, orthopedics, and general surgery.

This study was retrospective and used an unblinded and uncontrolled database with permission from the Institutional Review Board. MSB was mandated from January 1, 2003 onward. However, a historical control period, 1 January 2001–31 December 2003, was established for comparison (2003 was included because use of MSB alone did not change the CRBSI rate that year) with the years of intensivist supervision (2004–2007). There were seven requirements of MSB technique: hand washing before line placement; sterile site preparation; draping of the entire patient in sterile fashion; use of hat, mask, gloves and gown; maintenance of a sterile field; assistants following the same precautions; and sterile dressing application. Chemically impregnated catheters were not used. Chlorhexidine and Biopatch™ were required in all cases. Access kits were manufactured by Arrow International (triple lumen catheter kits, AK-09903-S; introducer sheaths, AK-22703). Ultrasound was available for CVC placements. CVCs were inserted only by residents on the critical care team. All residents were post-graduate years 2–6, and were either anesthesiology or surgery residents. The team of supervising intensivists included one anesthesiologist and two surgeons, all with special credential certification in critical care. The anesthesiologist provided 50% of the coverage and each surgeon provided 25% of the coverage. The Centers for Disease Control definition of CRBSI was used [7].

In calendar year 2003, with the institution of mandatory MSB use, the Infection Control (IC) department instituted a surveillance program to determine resident compliance with the MSB use in the SICU. There were 85 observed resident CVC placements in which the residents' MSB use was evaluated for technique breaks by independent observers. The decision of when to observe was arbitrary and occurred between 0700 and 1900 hours. Technique breaks occurred in 34/85 procedures and were associated with 6 CRBSI (17.6%). The 51/85 procedures without a technique break had 1 infection (1.9%), p < .01. The CRBSI rate for this initial year of MSB use was 5.0/1000 catheter-days (14 infections/2796 catheter-days); see Table 1.

**Table 1:** Differences in the CRBSI^a ^rates between the pre- (2001–2003) and post- (2004–2007)^b ^intensivist-led critical care team time frames

**Year**	**Catheter Days**	**Infections**	**Rate (%)**
2001	3065	18	.59
2002	2676	14	.52
2003	2796	14	.50
2004	3321	6	.18
2005	3304	7	.21
2006	3703	5	.14
2007	4005	3	.08

^a^Catheter-related blood stream infections.

^b^The Cochran-Armitage Trend Test indicated significant downward linear trend in the rates (Z = 4.576, p < .0001) for years 2004–2007.

From 2004 onward MSB use was augmented by an intensivist-led critical care team, in which intensivists were expected to actively participate in the oversight of SICU CVC placements. Notes were written on CVC placements and billed. Supervision of CVC placement, in addition to MSB use, resulted in a reduction of CRBSI to 0.8/1000 catheter-days (3 infections/4005 catheter-days) by 2007; see Table 1. With the institution of intensivist supervision in 2004 the IC Department discontinued their resident observations for technique breaks.

The Cochran-Armitage Trend Test was used for statistical analysis of annual trend and proportion of CRBSI. Also, an Arima Time Series Analysis was done for comparison of the monthly rates of infection [8]. Power calculation regarding sample size yielded a β of 0.999. A P-value less than .05 was considered statistically significant.

The analysis for annual trend and proportion of CRBSI demonstrated a significant downward linear trend between the years before the expectation of intensivist supervision of resident-placed CVCs (2001–2003), and after such an expectation was a matter of policy (2004–2007), Z = 4.576, p < .0001 (Table 1). The time series analysis comparing monthly CRBSI rates also revealed a downward trend, p = .028.

Our SICU CRBSI incidence often exceeded the national rate and caused the institution to demand MSB use. When the critical care service was organized, intensivists were instructed that there was an expectation of their presence at resident CVC placements. The intensivist was expected to correct resident technique and assist with CVC placement if necessary. However, the number of CVCs that intensivists had to place themselves, ultrasound device usage, or the attempts needed to place a particular catheter cannot be confirmed.

The added expertise and supervision afforded by intensivists facilitated greater scrutiny of technique and created a more consistent teaching/mentoring environment for residents. Some authors have described educational interventions that have demonstrated only a modest effect in compliance with "best practices" [9]. Coopersmith et al showed that, although educational programs can decrease CRBSI, periodic behavioral interventions are required to reinforce prior instruction and discourage reintroduction of previous bedside deficiencies [10]. Compliant resident behavior can be reinforced by the presence of a critical care physician. Furthermore, peer pressure from a cadre of resident critical care providers who are also held to standards of excellence through mentors' observations and by epidemiologic surveillance techniques may result in a superior result for patients.

Implementation of mandatory MSB use may reduce CRBSI incidence, but the intended result may be defeated if sterile technique is not meticulously followed. The IC department's 2003 resident surveillance for compliance demonstrated that breaches in technique carried nearly a nine-fold increase in risk. The presence of a knowledgeable and authoritative observer is very likely to have an impact upon the strict adherence to technique and thus may further decrease already low rates of infection. The maturation effect on the technique utilized by the individuals placing CVCs is less likely an explanation for the decrease in the infection rates due to continual rotation of new personnel onto the critical care service. It is noteworthy that during the years examined in this report the severity of illness index increased in our SICU population while the medical diagnostic categories demonstrated no variation [11].

MSB use in CVC placement can lead to a savings of $68,000 with seven CRBSI and one death averted for every 270 catheter insertions, especially for non-emergent placements in hospital patients [2]. Nonetheless, physician compliance with MSB has been poor [12]. This may be secondary to physician preference not to use MSB, local practice standards, the inability of the scientific literature to convince physicians, or lack of physician awareness of data supportive of MSB. Young et al have demonstrated that a systems-based intervention can lead to a sustained decrease in CRBSI instead of relying on voluntary changes in physician behavior [13]. Such a sustained change may save $10,000 per CRBSI [13]. Despite data demonstrating decreased mortality and financial savings less than one half of Non-Veterans Administration Hospitals use maximal barrier precautions, chorhexidine, and avoidance of routine central venous catheter changes [14].

There are inherent weaknesses in this study. The study involved a retrospective analysis, and was not randomized. The principle, inherent difficulty in this study design is the problem of comparability of a consecutive baseline intervention. There could have been changes in the patient population, treatment, or environmental biases. For instance, alterations in antibiotic regimens may influence the rates of CRBSI by prophylactic use and/or through selective pressure of treatment approaches. Also, our baseline period only lasted 12 months (that of MSB use without intensivist supervision), followed by an intervention period of over three years. This could introduce bias by the incomparability of the observational periods, e.g., seasonal effects. Additionally, although intensivist supervision was expected, we cannot state the percentage of central catheter placements in which they were present, and neither can we account for the number of such catheters placed over the years. However, there were no other changes during the periods of observation that otherwise could have impacted the incidence of CRBSI, such as the type of hub used on central venous catheters, change in the nurse-to-patient ratio in the SICU, or change in blood culture practices, etc. Finally, a legitimate question can be raised as to whether such results can be achieved by simply implementing an intensive education program followed by a checklist used by nurses who are empowered to abort a procedure if breaks in aseptic technique are observed.

Nonetheless, these data support the value of intensivist supervision of residents placing CVC catheters in critically ill patients, at least initially, in that the intensivist's presence may have led to a "Hawthorne effect" [15], or established a higher level of care or performance that was accepted as the standard. In this study the primacy of MSB use could not be established. While MSB use is extremely important and indispensable, proper sterile technique may be of even more importance. Teaching and mentoring in regard to MSB use and CVC placement technique, with continuous reinforcement, were effective in lowering our SICU CRBSI incidence. Further primary, randomized studies need to be conducted to unequivocally establish the importance of MSB, and/or intensivist supervision, in CVC placement for CRBSI prevention.

## Competing interests

The authors declare that they have no competing interests.

## Authors' contributions

TJP, SJH, JMD conceived the project; TJP, MJB, and JJF cared for the patients; SAK performed the statistical analysis; and TJP, SJH, JMD, JPH, SAK, MJB, and JJF all participated in writing the manuscript.
